# 10-year follow-up study on attendance pattern after dental treatment in primary oral health care clinic for fearful patients

**DOI:** 10.1186/s12903-021-01869-6

**Published:** 2021-10-13

**Authors:** Taina Kankaala, Heikki Laine, Marja-Liisa Laitala, Päivi Rajavaara, Hannu Vähänikkilä, Paula Pesonen, Vuokko Anttonen

**Affiliations:** 1 Department of Cariology, Endodontology, and Pediatric Dentistry, Research Unit of Oral Health Sciences, University of Oulu, Oulu, Finland; 2 Dental Teaching Unit, City of Oulu, Finland; 3 Medical Research Center, University of Oulu and Oulu University Hospital, Oulu, Finland; 4 Biostatistician Infrastructure for Population Studies, Faculty of Medicine, University of Oulu, Oulu, Finland

**Keywords:** Dental fear, Dental anxiety, Dental attendance, Dental fear treatment

## Abstract

**Background:**

Dental fear may lead to avoidance of regular dental treatment. The scope of this long-term practe-based study was to monitor the dental attendance of patients who received chair-side dental and fear treatment.

**Methods:**

In 2000–2006, patients in the City of Oulu, Finland, received treatment for dental fear in the Clinic for Fearful Dental Patients (CFDP) from primary health care dentists trained on this subject. Of the originally treated patients (n = 163), 152 (93%) with sufficient information in dental records made up the study population. Information on their age and sex was available. The number of dental examinations, emergency visits and missed appointments was collected covering the follow-up period of 10 years 2006–2016. For analyses, data were dichotomized according to age at baseline and preliminary outcome baseline condition of dental fear treatment evaluated in 2006. To investigate association further, Poisson regression as well as binary logistic regression models were conducted. As register keeper, the City of Oulu gave permission for this retrospective data-based study.

**Results:**

Patients receiving dental fear treatment at younger age (2–10 y) had significantly more dental examinations than those treated at > 10 years. Preliminary success was associated with the number of examinations, but not with emergency visits and missed appointments. Sex was not a significant factor in later dental attendance. There was an association between few dental examinations and dental emergency care need with unsuccessful baseline outcome of dental fear treatment.

**Conclusions:**

Successful dental fear treatment especially at an early age is beneficial for future dental attendance measured by the number of examinations and consequently, less need for emergency care than in the opposite case. Successful fear treatment has positive impact on later dental care and regular dental attendance.

## Background

In developed countries, dental fear or dental anxiety is one of the most common fears and its harmful associations for dental health and attendance are well-known [1–4]. Among other things, dental fear associates with avoidance behaviour, inferior self-care, and oral health problems in general [5–8].

The difference between pathological, abnormal and permanent fear and normal, life-preserving, healthy fear is very small [6, 9]. The signs of fearful behaviour may range from facial expressions to even freezing or fleeing [10]. All these reactions may be activated while attending dental care. Avoidance of dental appointments may indicate avoidance of the fearful situation.

Fear can be described as a dynamic process unfolding over time rather than an on–off response to stimuli [9]. This implies that it is possible to modify the fear response by psychological means such as cognitive behavioural therapy (CBT) [11, 12]. Patients who have received cognitive behavioural therapy (CBT) for dental fear have reported a significant, in most cases permanent decrease in their fear to such a level that it does not disrupt later dental care [6, 13]. Indeed, successful reduction of dental fear may lead to regular dental attendance and the acceptance of normative dental care [11, 14–16], which in turn diminishes the need for emergency visits. However, reduction of dental fear does not always have a significant impact on dental attendance [17, 18]. There is a need for long-term follow-up after dental fear treatment to find successful ways to manage dental fear.

Management of dental fear with methods such as CBT is provided in primary oral health care in the City of Oulu, Finland, by dentists interested in this area. The clinic (Clinic for Fearful Dental Patients, CFDP) focusing on dental fearful referral patients was founded in 2000. The aim of dental fear treatment at the clinic is to alleviate dental fear to such a level that subsequent treatment can be administered in normal dental care.

The aim of this retrospective, practice-based follow-up study was to assess the long-term or 10-year outcome of dental fear treatment in the Clinic for Fearful Dental Patients (CFDP) in the City of Oulu, Finland, as indicated by the frequency of dental examinations, emergency visits and missed appointments. The hypothesis was that dental attendance is positively influenced by successful dental fear treatment regardless of patient’s age and sex at the time of the fear treatment.

## Methods

### Study population

**Fig. 1:**
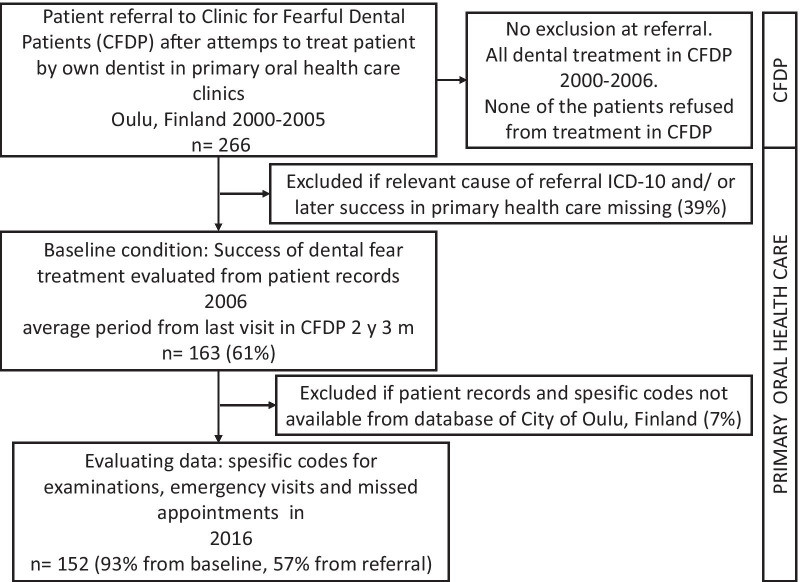
The participant flow chart. Number and proportions of full patient records evaluated

Patients in this retrospective, practise-based study had been referred from the primary oral health care clinics in the City of Oulu Public Dental Services (PDS) to the CFDP during the period 2000–2006 for dental and dental fear treatment. Referrals were sent by dentists working in primary oral health care after several unsuccessful attempts to treat the patients. The age of the patients or their medical condition was not an obstacle for referral, but the referring dentist had to give a relevant diagnose for referral and sufficient information of dental treatment need. The median age of the patients at the time of referral to dental fear treatment was 7 years (SD 7.3, min 2, max 51). The outcome of the baseline dental fear treatment in CFDP of 163 patients was evaluated in 2006 according to dental records in primary oral health care, later the term *baseline condition* is used. All referred patients had dental fear at referral: dental fear was not measured in the CFDP, but all were diagnosed based on detective clinical evaluation suggested by Milgrom et al. [19]. Baseline condition was determined based on later co-operation in primary dental care. The baseline condition outcome was considered successful in 2006 if no mention of fear or need for sedation or dental general anaesthesia (DGA) was discovered. Baseline condition success rate was 69% of the referred cases. If dental fear, lack of co-operation, sedation, DGA or second referral to CDFP was recorded in the patient files, the baseline condition was considered not successful. For this study, the patient records of a total of 152 of the 163 patients in 2006 were found in the patient database of the City of Oulu. The dates of all dental examinations, emergency visits and missed appointments after dental fear treatment were recorded for each patient. The patient flow is illustrated in Fig. 1.

### Setting

Patients were treated in CFDP by three dentists specially trained in treating fearful dental patients and with long clinical experience. CFDP is an integrated part of primary oral health care in PDS setting treating those referral dental patients of the Municipality of Oulu Finland, who could not cope in their own dental offices due to immaturity for dental treatment, behavioral management problems, gagging, generalised fear or fear of dental treatment, needles or procedures. Two of the dentists in CFDP are clinical practitioners and lecturers on the topic for students and one is a hypnotherapist. At the baseline, for all patients, any psychological, but suitable approach (CBT, desensitization, relaxation, distraction, or combination) was the main tool for treating dental fear at CFDP. No patients were excluded due to age or medical condition, for example. A psychologist or psychotherapist was consulted if needed. Additionally, conscious sedation was used when considered necessary. Because the referred patients had need for dental treatment as well (most commonly restorative treatment 87.7%, extractions 4.9% and orthodontic treatment 1.2%) all the dental procedures were also performed at CFDP. After dental and fear treatment in CFDP, patients were treated by their own dentists in PDS or by private dentists according their or their guardians choice. This description of the study method partly includes text from our previous study [20]. By public dental health setting dentists coded patients` examinations, emergency visits and missed appointments and data were readily available from the local patient record database. Those patients` full patient records (n = 9) who had moved to another area or continued their dental care in private dental offices were missing and were excluded from the study.

### Statistics

The data were collected in May 2016 from the patient files of the City of Oulu and prepared for analyses. The study population was dichotomized according to age (2–10 and > 10 years) and according to success of the original dental fear treatment evaluated in 2006 (baseline condition). The number of dental examinations, emergency visits, and missed appointments was recorded for each patient from 2006 baseline condition to 2016. These figures were categorised as follows: 0, 1–5, 6–10 and > 10 instances of the procedure of interest. The associations between the baseline condition and independent variables were analysed using cross tabulation and chi-square test.

A Poisson regression analysis was conducted to investigate the association between the number of dental examinations, emergency visits and missed appointments during the follow-up period 2006–2016 (95% CI) in association with baseline condition success in 2006, sex of the patients, as well as age at the time of the referral.

Binary logistic regression models were conducted to present odds ratios and their 95% confidence interval (OR, 95% CI). The baseline condition outcome of the treatment in 2006 was used as dependent variable (success/not success) separately for the age groups at the time of the dental fear treatment with the cut-off point of 10 years (2–10/> 10 y). As independent continuous variables were the number of dental examinations, emergency visits and missed appointments. The two-way interaction terms have also tested and the models have adjusted with statistically significant interaction term of examinations and emergency visits. A separate logistic regression model was conducted among those with 0–5 examinations/> 5 examinations).

*p* values of *p* < 0.05 were considered statistically significant, after Bonferroni adjustment 0.05/8 = 0.006. All analyses were executed with the SPSS (version 26.0, SPSS, Inc., Chicago, IL, USA).

## Results

The patient files of n = 152 patients were analysed in 2016, i.e. 93% of those whose baseline condition of dental fear treatment from 2006 and full records were available in the database of PDS. A total of n = 2591 registered procedures accomplished within the 10-year period from 2006 to 2016 were investigated. At the baseline referral 2000–2005, the mean age of the participants was 8.9 years (median 7 y, SD 7.3, min 2, max 51). Majority (79.6%) of the participants were 2–10 years old during the fear treatment in CFDP 2000–2006. In 2016 mean age of the patients with was 21.7 years (median age 20 y, SD 7.3, min 14, max 64). The proportion of male participants (n = 89, 58.6%) was higher than that of females (n = 63, 41.4%). Twenty-nine patients (n = 29, 19%) were mentally or medically compromised, others were healthy. The median of dental examinations was higher among those treated at the age of 2–10 y (9, min 0, max 21) compared with those treated > 10 y (4, min 0, max 11), whereas the median of emergency visits was similar in both age groups (4, min 0, max 28 vs. 5, min 0, min 29). The number of missed appointments also varied greatly in both age groups (2, min 0, max 20; 1 min 0, max 15, respectively). The proportion of those with at least six dental examinations was distinctly higher among the ones treated in early years compared with those treated at the age > 10 y (*p* = 0.001), this result remains significant also after adjusted with Bonferroni correction. (Table 1). Over half (58.1%) of the patients older than 10 years at the baseline had 1–5 dental examinations during the observation period and only one of them (3.2%) had > 10 examinations whereas the respective proportion for the younger ones was tenfold. Only three patients missed their dental examinations completely during the follow-up period (Table 1).

**Table 1:** Number of dental examinations (A), emergency visits (B) and missed appointments (C) after dental fear treatment categorized by age at the time of treatment

	n (%)					
	0	1–5	6–10	> 10	Total	*p* value*
(A) Number and proportion of examinations						
Age						
2–10	2 (1.7)	31 (25.6)	50 (41.3)	38 (31.4)	121	0.001
> 10	1 (3.2)	18 (58.1)	11 (35.5)	1 (3.2)	31	
Total	3 (2.0)	49 (32.2)	61 (40.1)	39 (25.7)	152	
(B) Number and proportion of emergency care visits						
Age						
2–10	10 (8.3)	66 (54.5)	30 (24.8)	15 (12.4)	121	0.990
> 10	2 (6.5)	17 (54.8)	8 (25.8)	4 (12.9)	31	
Total	12 (7.9)	83 (54.6)	38 (25.0)	19 (12.5)	152	
(C) Number of missed appointments						
Age						
2–10	24 (19.8)	72 (59.5)	18 (14.9)	7 (5.8)	121	0.673
> 10	9 (29.0)	15 (48.4)	5 (16.1)	2 (6.5)	31	
Total	33 (21.7)	87 (57.2)	23 (15.1)	9 (5.9)	152	

*Chi-square test

Males had more dental examinations than females (n.s.). Nearly three quarters (70.8%) of males had six or more examinations during the observation period while less than two thirds (58.7%) of females fell in this category (Table 2). A preliminary successful outcome in 2006 as a baseline condition associated with an increased number of examinations (*p* = 0.047), except that those with no success at baseline had more frequently more than > 10 examinations. With a preliminary successful baseline condition, the proportion of those having 6–10 examinations was larger during the following 10 years compared with the situation when the baseline condition was not successful (46.7% vs. 24.4%), (Table 3).

**Table 2:** Number of dental examinations (A), emergency visits (B) and missed appointments (C) after dental fear treatment categorized by sex

	n (%)					
	0	1–5	6–10	> 10	Total	*p* value*
(A) Number and proportion of examinations						
Sex						
Female	2 (3.2)	24 (38.1)	22 (34.9)	15 (23.8)	63	0.047
Male	1 (1.1)	25 (28.1)	39 (43.8)	24 (27.0)	89	
Total	3 (2.0)	49 (32.2)	61 (40.0)	39 (25.7)	152	
(B) Number and proportion of emergency care visits						
Sex						
Female	4 (6.3)	37 (58.7)	18 (28.6)	4 (6.3)	63	0.215
Male	8 (9.0)	46 (51.7)	20 (22.5)	15 (16.9)	89	
Total	12 (7.9)	83 (54.6)	38 (25.0)	19 (12.5)	152	
(C) Number of missed appointments						
Sex						
Female	12 (19.0)	40 (63.5)	10 (15.9)	1 (1.6)	63	0.209
Male	21 (23.6)	47 (52.8)	13 (14.6)	8 (9.0)	89	
Total	33 (21.7)	87 (57.2)	23 (15.1)	9 (5.9)	152	

*Chi-square test

**Table 3:** Number of dental examinations (A), emergency visits (B) and missed (C) after dental fear treatment categorized by initial dental fear treatment success at baseline 2006

	n (%)					
	0	1–5	6–10	> 10	Total	*p* value*
(A) Number and proportion of examinations						
Baseline success						
Unsuccessful	2 (4.4)	17 (37.8)	11 (24.4)	15 (33.3)	45	0.047
Successful	1 (0.9)	32 (29.9)	50 (46.7)	24 (22.4)	107	
Total	3 (2.0)	49 (32.2)	61 (40.1)	39 (25.7)	152	
(B) Number and proportion of emergency care visits						
Baseline success						
Unsuccessful	1 (2.2)	23 (51.1)	16 (35.6)	5 (11.1)	45	0.127
Successful	11 (10.3)	60 (56.1)	22 (20.6)	14 (13.1)	107	
Total	12 (7.9)	83 (54.6)	38 (25.0)	19 (12.5)	152	
(C) Number of missed appointments						
Baseline success						
Unsuccessful	7 (15.6)	31 (68.9)	5 (11.1)	2 (4.4)	45	0.314
Successful	26 (24.3)	56 (52.3)	18 (16.8)	7 (6.5)	107	
Total	33 (21.7)	87 (57.2)	23 (15.1)	9 (5.9)	152	

*Chi-square test

In the age group 2–10 y, the median number of emergency visits was 4 (min 0, max 28) while the corresponding figure for those in the age group > 10 y was 5 (min 0, max 29). More than half of the patients had had 1–5 emergency visits, whereas over one third had had more than five emergency visits. On the other hand, almost one in ten had had no emergency visits (Table 1). There was no statistically significant difference in the number of emergency visits in terms of age at referral or sex (Table 2).

The number of patients with no emergency visits was considerably higher in the baseline condition success group compared with the group with no success in dental fear treatment (n.s.). Only one patient (2.2%) in the unsuccessful fear treatment baseline condition group avoided emergency treatment while the proportion in the successful group was 10.3% (Table 3).

In the age group 2–10 y, the median number of missed appointments was 2 (min 0, max 20) while the corresponding figure for those in the age group > 10 y was 1 (min 0, max 15). Almost half of the patients fell into the category 1–5 missed appointments; no statistically significant difference was discovered between the age groups. Nine of the patients had failed to show up more than 10 times (Table 1). Interestingly, all but two belonged to the age group 2–10 y at referral (Table 1), eight of them were males (Table 2). There seems to be little or no correlation between the success in dental fear treatment at baseline and the number of missed appointments (n.s., Table 3).

According to Poisson regression models only young age at referral was statistically significantly associated with future increased number of dental examinations (λ 0.687, 95% CI min 0.423, max 0.951). Other associations were not discovered. In the binary logistic regression model in the younger age group, no statistically significant association was discovered between the dental attendance variables and preliminary success of dental fear treatment. In the older age group, however, emergency visits decreased the odds for preliminary success (OR 0.15; 95% CI 0.033, 0.685) and on the other hand, the combined effect of examinations and emergency calls was positively associated with preliminary success (OR 1.285; 95% CI 1.044, 1.583). Among those with 0–5 examinations, even one emergency call was associated with a decreased risk of preliminary success of dental fear treatment (OR 0.269; 95% CI 0.084, 0.856); this was not seen among those with more than five examinations during the monitoring period.

## Discussion

The aim of this retrospective, data-based 10-year follow-up study was to assess the effect of dental fear treatment in the Clinic for Fearful Dental Patients (CFDP) in the City of Oulu, Finland, on dental attendance in primary health care as indicated by examinations, emergency visits and missed appointments. During the 10-year-follow-up, those who had been referred to dental fear treatment at an early age (< 10 years) or whose dental fear treatment was considered successful in 2006 [20] had more dental examinations than the rest. As for emergency visits and missed appointments, no association with the success of dental fear treatment was discovered. Among those older than 10 years at the baseline and with 5 or less examinations during the follow-up period, even one emergency visit indicates that the preliminary dental fear treatment outcome in 2006 baseline was not successful.

The patient sample was relatively small and patients of only one specialized unit for dental fear treatment were observed. However, the outcome could be monitored for 57% of the originally treated individuals and 93% of those for whom the short-term baseline outcome was investigated in 2006. Heterogeneity in terms of age in the study population can be considered a shortcoming. The biggest difference between a similar study by Berge et al. [12] and the current one is that here, all patients were included despite their physical or mental status or the dental care they needed. In addition, all the dental procedures needed were performed during dental fear treatment, including dental general anaesthesia as well. Considering the challenges in the study population, the 2- and 10-year outcomes seem satisfactory. On the other hand, the follow-up period in this study from early childhood to adolescence or even adulthood was longer compared to many other studies [17, 18]. Participants success of dental fear treatment as a baseline condition was evaluated in 2006 and has not been evaluated in a similar manner later on. So, it is possible, that in some cases the outcome was positive after 2006, but also some positive outcomes may have been negative later on. However, this work aims to find the longterm impact of dental fear treatment. Length of the monitoring period for some of the participants was even longer than 10 years, which can be considered a strength.

The benefit here is a long monitoring period of 10 years; to our knowledge, this is the only study of its kind in a public dental health setting. In earlier studies, patients were monitored for shorter periods [13, 18] or two separate cohorts were compared [21], or dental fear treatment was given by a psychotherapist [11, 16]. It is also common to use surveys in studies of this kind and the response rates tend to be quite low [17, 22]. Patients with severe dental fear may not participate in such surveys if they avoid dental appointments in general [18]. Data based on patient records, not dependent on participation, is a benefit in a study like the present one, where the study population can be monitored with or without attendance. The Finnish Public Dental Services provides reliable longitudinal data for research purposes [23]. The present study population was 152 patients who made up 93% of the population evaluated in 2006 for the success of dental fear treatment with a follow-up period of about 2 years. Most participants were children living at home at the time of the original dental fear treatment (2000–2006) and still lived in Oulu in 2016 (93%) [20]. Because the lack of nationwide welfare databases before year 2017 records of those patients`who have moved to another area or continued their dental care in private dental offices were missing. This is a shortcoming in this database study.

Irregular or non-existent dental attendance because of dental fear is associated with a vicious circle of dental care avoidance, fear and poor oral health [1, 2, 24]. Avoidance causes suffering for individuals, and expenses for them as well as for society [25]. Finnish dental care system recommends examinations according certain intervals, which explains fewer examinations among the youngest participants. Individual intervals should be shorter if a patient has treatment needs or is at high risk i.e. for dental caries. At the referral all patients had a need for dental treatment: 92.6% had cariological problems or needed extractions which may indicate that they are at risk also for deteriorating oral health if not treated regularly. Thus, despite age, they should have had recall periods (examinations), maximum 1 year. It appears that males had more dental examinations than females. Those with a successful baseline condition had more dental examinations than the ones with no success, which can be considered a sign of regular dental attendance among those with preliminary success. This result is in accord with the findings of the review by Wide-Boman et al. [15].

Dental fear, if not addressed, can persist and complicate life over the years. Adolescents are in their sensitive years of life due to physical as well as mental developmental changes and are at risk of anxiety disorders later in life if the fears are not treated [16]. Children under 10 years have not usually reached their adolescence phase with rapid changes in cognitive and psychological development towards adulthood [26], which may affect the outcome of dental fear treatment and this was the cut-off point of the present dichotomization. The small size of the study population hindered comparing several age groups instead of two. Here, those who belonged to the age group of > 10 years at the time of dental fear treatment tended to have more emergency visits than the ones treated at a younger age. This shows that it is more challenging to have a positive effect on the vicious circle after early childhood years. Males tended to have a higher number of emergency visits (> 10) than females, as has been reported in previous literature [27–29]. Details of the clinical situation at the baseline were not available but may have caused the difference.

Our previous study indicated that it is beneficial to treat dental fear at a young age, and the same trend is seen here. Preliminary successful baseline outcome of dental fear treatment was specifically associated with optimal number of dental examinations, which can be considered a sign of regular dental attendance among those with preliminary success. This study indicated that there is an association between dental attendance with a low number of examinations with at least one dental emergency visit and not successful preliminary dental fear baseline condition. It is essential to recognize and bring to regular dental care this vulnerable group, whose dental attendance and avoidance can be influenced by fear treatment. Emergency dental care could be a place for recognizing this group by using dental fear forms, for instance. Research is needed on this topic.

The number of missed appointments per se was fairly low in both age groups, but varied a great deal. The proportion of those with more than 10 missed appointments was 5.9% while the proportion among boys was 9.0% and among girls 1.6%. Male sex seems to be associated with avoidance, as also seen in literature [30]. The figures for all missed appointments were higher here than for general population in the City of Oulu, Finland; 3–5% at the time of the study (Statistics, the City of Oulu Finland 2019) and among those having at least one missed appointment in line with a previous study of Tilja et al. on DGA patients [31]. In our study there was a trend that a successful preliminary baseline condition was associated with no missed appointments.

It has been reported that reduction in dental fear measured by surveys does not necessarily correlate with improved dental attendance [17]. Dental fear surveys are essential in detecting, treating and monitoring dental fear in general practice. Here, the preliminary reduction of dental fear was practice-based, based on successful dental attendance in primary health care after dental fear treatment. The present protocol appeared to be beneficial—the preliminary success of dental fear treatment and later dental attendance were associated. Despite the fairly high preliminary success of dental fear treatment, this study population remains a challenge and special attention should be given to regular recalls of fearful patients, especially as most negative dental treatment experiences may lead to the reactivation of dental fear despite positive former experiences [32]. In the future, similar practice-based studies can reveal the patterns of dental attendance, e.g. after successful dental fear treatment.

## Conclusion

To conclude, this practice- and data-based study suggests that an individual approach chair-side to dental fear can be effective in bringing patients to regular, examination-based oral care indicated by a monitoring period of 10 years after dental fear treatment. This was also our hypothesis. Our results are also in line with our second hypothesis concerning sex, but contradictory for age at the time of dental fear treatment. Administering dental fear treatment is beneficial when done at an early age, as the results then, seem to be more persistent. Preliminary baseline success was not significantly associated with emergency visits and missed appointments. However, there seems to be an association between not successful baseline condition of dental fear treatment and a low number of dental examinations combined with the use of dental emergency care. Successful fear treatment has positive impact on later dental care and regular dental attendance.

## Data Availability

The data are kept in the University of Oulu until and reasonable requests can be directed to the authors.

## Abbreviations

CFDP: Clinic for Fearful Dental Patients
CBT: Cognitive behavioural therapy
CI: Confidence interval
DGA: Dental general anaesthesia
PDS: Public Dental Services
SD: Standard deviation
SPSS: Statistical Package for the Social Sciences
